# Characteristics and Treatment Results of 5 Patients with Fibrous Dysplasia and Review of the Literature

**DOI:** 10.1155/2015/670809

**Published:** 2015-06-14

**Authors:** Nilufer Ozdemir Kutbay, Banu Sarer Yurekli, Emine Kartal Baykan, Serap Baydur Sahin, Fusun Saygili

**Affiliations:** ^1^Endocrinology Department, Ege University Faculty of Medicine, Izmir, Turkey; ^2^Endocrinology Department, Recep Tayyip Erdogan University Faculty of Medicine, Rize, Turkey

## Abstract

*Aim*. Fibrous dysplasia is a rare bone disease caused by missense mutation leading to abnormal fibroblast and osteoblast proliferation and increased bone resorption. FD can present in monostotic or polyostotic forms. About 3% of FD could be in association with McCune-Albright syndrome (MAS). Because FD is a rare disease, there is limited data in the literature about characteristics of disease and response to treatment. *Methods*. We present our five cases of FD with general properties and their responses to medical treatment. *Results*. Two of our patients had polyostotic and three had monostotic FD. One of the polyostotic patients had MAS. One of our patients had surgery for femur fractures, facial asymmetry, and findings of compression. Four patients were given pamidronate; one was given zoledronic acid as bisphosphonate treatment. Bone pain was relieved in all patients with medical treatment. *Conclusion*. There was a decrease in bone turnover markers to some degree with medical treatment but no radiological improvement was observed.

## 1. Introduction

Fibrous dysplasia (FD) is a benign bone disease which has genetic noninheritable origin. FD occurs due to missense mutation in gene coding for the *α* subunit of the stimulatory G-protein, Gs, in the GNAS complex locus in chromosome 20q13. As a result of these GNAS mutations, abnormal proliferation and differentiation of bone marrow stromal cells lead to fusiform fibroblast-like cells. Those cells create poorly differentiated osteoblasts [1]. Abnormal bone in a fibrous stroma results in increased bone resorption with the hyperactivity of osteoclastic cells [2].

FD is a rare bone disease leading to bone pain, deformity, and fracture. It is common that FD could be a silent disease. FD was first described by Lichtenstein in 1938 [3]. It has been estimated that the prevalence may be around 1/30.000 [4]. Men and women are equally affected [2]. It is benign, slowly progressing disease and especially involving craniofacial skeleton, long bones, and costa [5]. FD can present in monostotic form with one bone site or in polyostotic form with multiple bone sites. Monostotic forms compromise 70–80% of FD; rest of FD mostly consists of polyostotic forms [2].

Many patients have limited bone involvement with no symptom or bone pain, but the disease can cause significant disabilities like bone deformity and neurologic compressions [2]. Some patients have endocrine abnormalities and hyperpigmentation of the skin so-called McCune-Albright syndrome (MAS) [6].

There is no spontaneous resolution of FD. Not all patients require treatment. Surgery may be indicated if bone deformity is large and causes compression of adjacent tissue. Bisphosphonates are often used as medical treatment as they may reduce the increased bone resorption [6]. Because of the fact that FD is a rare disease, a few published data are available. Herein, we present our five cases of FD with clinical properties and medical treatment results.

## 2. Materials and Methods

We identified five patients with FD at our Endocrinology Department, Ege University Hospital, Turkey. We collected data on gender, age, symptoms, and findings at presentation. We examined the radiographic images of our patients with FD. In addition, we gathered data about medical treatment and response to the treatment as relief of symptoms and as biochemical findings. Biochemically, serum alkaline phosphatase (ALP), serum osteocalcin (OC), and deoxypyridinoline (DPD) in urine were measured as bone turnover markers. In regard to bone, serum calcium (Ca), serum phosphor (P), and serum parathormone (PTH) levels were recorded. All data were collected retrospectively. Total serum ALP was measured with Roche Hitachi 797, Tokyo, and normal reference range was 40–129 U/L. Serum OC and DPD were measured with Immulite 2000, UK. Normal reference range for OC was 2–15 ng/mL and for DPD was 2.3–5.4 nmol/L. Plasma levels of intact parathyroid hormone (PTH) were measured using a second-generation electrochemiluminescence immunoassay. We present characteristics of our patients.

### 2.1. Patient 1

**Figure 1:**
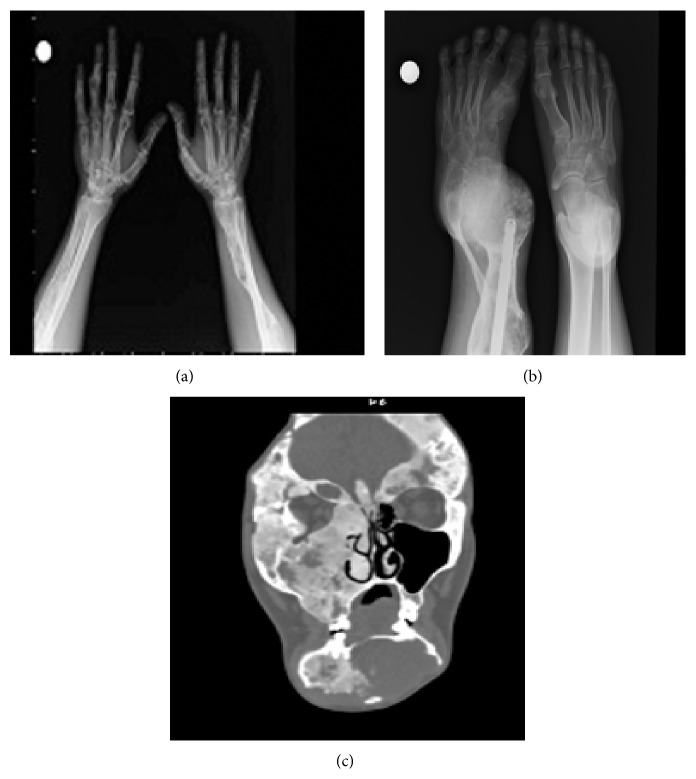
Radiologic images of patient 1. (a) X-ray of hands and forearms. (b) X-ray of feet. (c) CT images of craniofacial bones.

A thirty-one-year-old female patient was admitted to Emergency Department when she was two and a half years old. Right femur fracture was diagnosed at hospital. Recurrent fractures had occurred at left femur, tibia, and humerus. Femur biopsy that was performed 7 years ago revealed the diagnosis of FD. During the follow-up of the patient, deformity of facial bones evolved. Maxillofacial computed tomography (CT) revealed deformities on sphenoid bone. Those deformities were obliterating maxillary sinus and reaching to the zygomatic bone. Ethmoid sinuses and mandibular bones were also involved. Ground glass appearance was observed at the mandibular bones. On the right side of cranium, there was narrowing at the level of pterygopalatine fossa and foramen rotundum. There were findings of exophthalmic appearance at right orbita (Figure 1(c)). Lytic expansile lesions were seen at the right iliac bone. Bending was observed at the right femur. Also, along the femur, lesions compatible with FD were present. Bone scintigraphy showed that there was increase in osteoblastic activity at maxillofacial sites, right and left humerus, right and left femur and tibia, ischium, and 2nd and 4th costae. As a result of craniofacial involvement, she had facial deformity and bone pain, so operation was performed by neurosurgeon. Pamidronate was given as 60 mg IV on consecutive 3 days, a total of 180 mg. Bone turnover markers after bisphosphonate treatment were presented at Table 1. Bone pain subsided after medical treatment. There was no new fracture and progression of bone lesions during follow-up of a 7-year period.

### 2.2. Patient 2

A thirty-year-old male patient was admitted to the hospital, Department of Plastic Surgery, with the complaint of bone pain at mandible. Cranial CT showed that changes at mandible were compatible with fibrous dysplasia (not shown). FD was diagnosed 9 years ago. Pamidronate was given as 180 mg intravenously. Bone turnover markers before and after bisphosphonate treatment were shown in Table 1. He had no operation. Bone pain decreased after medical treatment.

### 2.3. Patient 3

**Figure 2:**
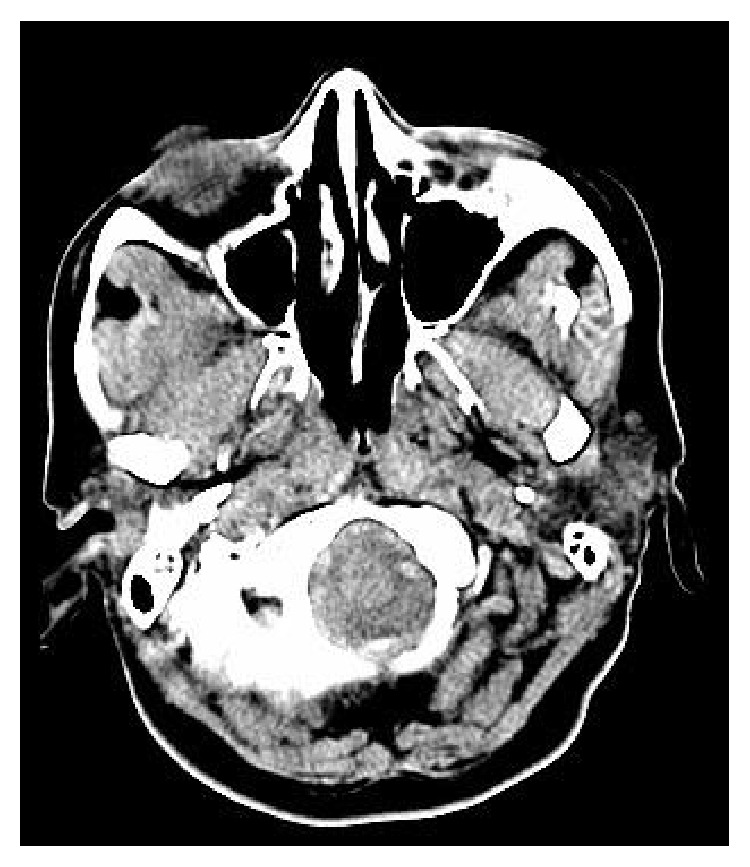
Cranial CT images of patient 3.

A fifty-three-year-old female patient had the complaint of headache four years ago. When cranial CT was performed there were lytic and sclerotic bone lesions at the right frontal bone. Those lesions were compatible with fibrous dysplasia (Figure 2). There was increased activity at cranium according to the bone scintigraphy. FD was diagnosed clinically with the aid of imaging modalities. Operation was not performed. Only medical treatment was recommended as zoledronic acid 5 mg intravenously. After zoledronic acid treatment, the complaints of the patient subsided. The changes in bone turnover markers with medical treatment were shown in Table 1.

### 2.4. Patient 4

**Figure 3:**
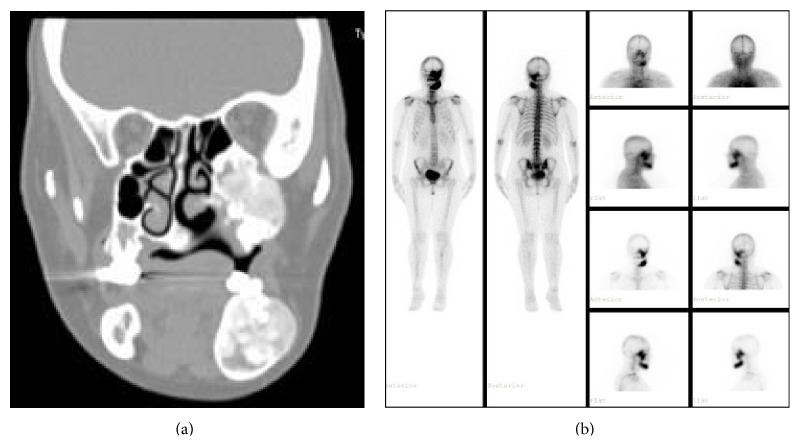
Radiologic and scintigraphic images of patient 4. (a) Cranial CT image. (b) Scintigraphic image.

A forty-three-year-old female patient was admitted to the hospital, Ear, Nose, and Throat Department, with the complaint of bone pain and facial deformity three years ago. Cranial CT showed expansion and deformity pointing to FD at the sites of sphenoid bone, left maxillary sinus, zygomatic bone, and mandibular bone (Figure 3(a)). As a result of these changes, there was narrowing at the inferior orbital fissure and pterygopalatine fossa. There was no aeration at the left maxillary and sphenoid sinuses (Figure 3(a)). Increased activity at maxilla and mandible was seen in bone scintigraphy pointed to FD (Figure 3(b)). Pamidronate of 180 mg total dose was given intravenously. Bone pain was resolved after treatment and changes in bone turnover markers were shown in Table 1. Bone lesions seen at CT were stable during follow-up.

### 2.5. Patient 5

**Figure 4:**
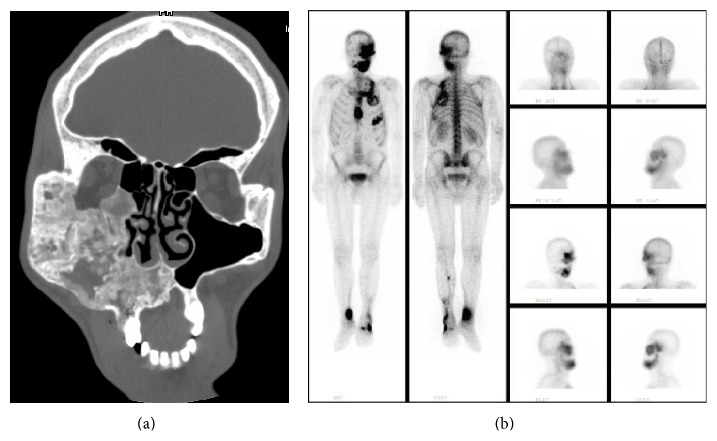
Radiologic and scintigraphic images of patient 5. (a) Cranial CT image. (b) Scintigraphic image.

A forty-one-year-old male patient has had diagnosis of FD for 20 years. FD was diagnosed according to maxillary lesion seen at cranial CT. He has also had diagnosis of acromegaly for 20 years. Pituitary surgery could not be performed because of the maxillary and sphenoid sinus lesion. There was scene of FD at the left temporal, sphenoid, maxillary, and mandibular bones causing expansion and changes in density. There was a contraction of superior and inferior orbital fissures (Figure 4(a)). As treatment of acromegaly, he was using octreotide and dopamine agonist. Radiotherapy for the acromegaly was performed 3 years ago. Bone scintigraphy showed increased activity at left maxilla and mandible, sternum, and 1st, 5th, and 6th costae (Figure 4(b)). Changes in bone turnover markers after 180 mg pamidronate treatment were presented in Table 1. Together with acromegaly, it was thought that he had atypical presentation of MAS, because he had no café-au-lait macules.

In the literature there was a report of case series presenting as atypical MAS [7]. McCune-Albright syndrome (MAS) is a rare disease which is characterized by the three features: fibrous dysplasia (usually polyostotic), café-au-lait macules, and endocrine hyperfunction [7]. Endocrinopathies often include sexual precocity, hyperthyroidism, hypercortisolism, GH excess, and hyperprolactinemia. One or two classical symptoms of MAS were present in atypical MAS. GNAS mutation analysis was present in <50% of patients with classic triad of MAS. Therefore, the use of mutation analysis in those patients is limited and diagnosis of atypical MAS remains challenging [7]. In our patient GNAS mutation was not detected.

## 3. Results

We identified a total of 5 patients (*n* = 3 F; *n* = 2  M). Mean age of our patients was 39.6 (30–53 years) years old. The clinical characteristics of our patients were shown in Table 2. FD was diagnosed histopathologically in our two patients (P1 and P5). It was diagnosed radiologically in other three patients (P2, P3, and P4). We had two cases with polyostotic fibrous dysplasia (PFD) and 3 cases with monostotic fibrous dysplasia (MFD) (Table 1). One of PFD patients had atypical presentation of MAS (P5). This patient had craniofacial involvement including temporal, maxillary, mandible, and sphenoid bones and costa involvement. The diagnosis of FD was verified by maxillary biopsy. The other PFD patient (P1) had also craniofacial deformities. She had a craniofacial reconstructive operation for the relief of neurologic compression.

**Figure 5:**
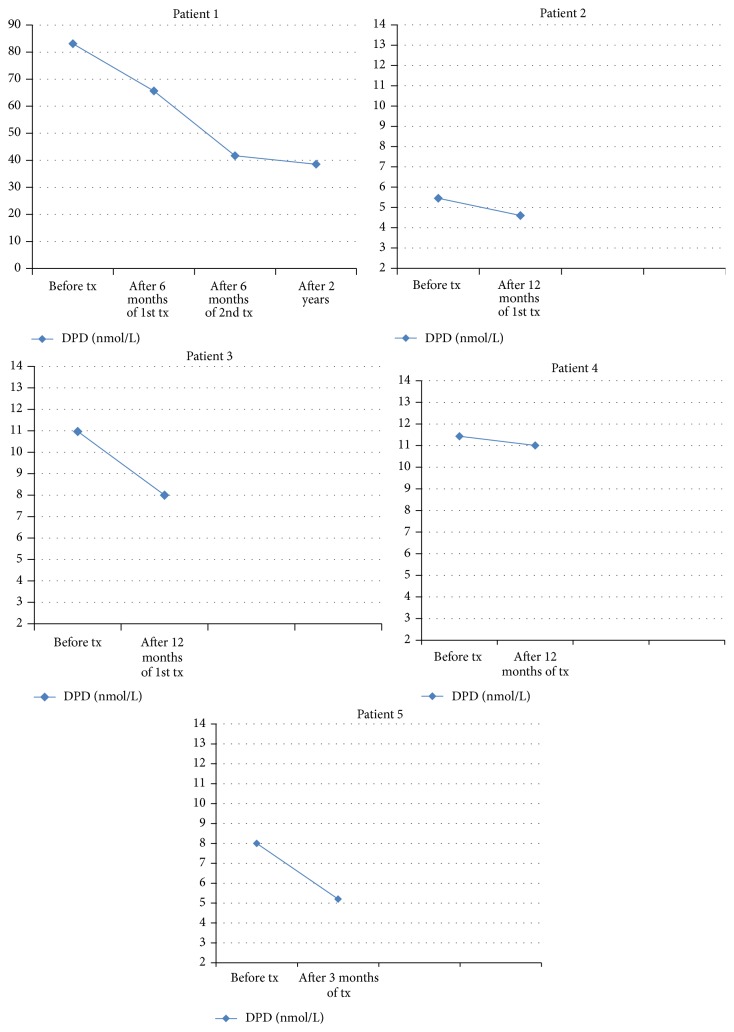
The changes in urinary DPD levels before and after bisphosphonate treatment.

Bisphosphonate treatment was preferred for five cases due to bone pain and compression. Three doses of pamidronate 60 mg IV (total dose 180 mg) were given to four cases, and zoledronic acid 5 mg IV was administered to one case (Table 1). P1 was given 180 mg pamidronate for two times. As far as bone turnover markers are concerned, DPD levels were decreased by 54.2%, 15.5%, 3.5%, and 35.0% in P1, P2, P4, and P5, respectively, with treatment of pamidronate. Meanwhile, 26.6% decrement in DPD levels was observed in P3 with treatment of zoledronic acid (Figure 5). All our patients were given calcium and vitamin D replacement to prevent secondary hyperparathyroidism. The complaints of pain were less with bisphosphonates treatment; however, no radiological change was observed. The bone markers of the patients before and after medical treatment were presented in Table 2.

## 4. Discussion

FD is a rare benign bone disease. As it can be asymptomatic, FD can also cause bone pain, deformity, and compression symptoms. When fibrous dysplasia is symptomatic, medical and/or surgical treatment could be an option for therapeutic modalities. All our patients were symptomatic and bisphosphonates as medical treatment were prescribed for all our FD patients.

Potent antiresorptive agents, bisphosphonates, were first used in 1990s [8]. In the study of Liens et al. in 1994, it was observed that there was a rapid improvement in radiologic appearance and in bone turnover markers after medical treatment [9]. Besides, there was relief in bone pain with the pamidronate treatment given as 180 mg (60 mg/day, consecutive 3 days) intravenously every 6 months [9]. It was seen that the major effect of bisphosphonate treatment was the decrease in bone pain. In our cases, all patients had relief in their bone pain. In the study of Parisi and colleague, seven patients were given pamidronate for a mean of 6.9 years (3.7–10.9 years) [10]. There was a significant decrease in serum levels of ALP and bone pain. CTX-1 levels decreased by 56% compared to the beginning of treatment. In our cases, the longest follow-up period was 24 months with patient 1. For other patients, mean follow-up period was 12 months. During this period, while there was a decrease in bone pain, there was no improvement in radiologic appearance. As far as bone turnover markers were concerned, DPD levels were decreased with treatment of pamidronate and zoledronic acid. Because of the small number of our cases, we cannot compare the effectiveness of two different bisphosphonate treatments.

There were no serious side effects in our patients taking pamidronate and zoledronic acid. In the literature, zoledronic acid (4 mg every 6 months) was given to 9 patients after pamidronate in case of relapse or no response. While there was 60% decrease in pain, serum CTX levels decreased by 24% with the treatment of zoledronic acid [8]. In this study, there was an acute phase reaction as a side effect in two patients [8]. It could not be possible to compare two bisphosphonates treatments because of the short follow-up period and lack of randomized studies. There is a need for long term studies to gather enough data about the usage of zoledronic acid in FD.

To the best of our knowledge, there was only one randomized clinical study regarding the treatment of FD in the literature. This study was a 2-year randomized, double-blinded, placebo controlled study of oral alendronate in FD. Alendronate was given as 40 mg/day for the patients >50 kg, 20 mg for 30–50 kg, and 10 mg for 20–30 kg over a 24-month period in 6-month cycles. Forty FD patients (24 adults and 16 children) were enrolled in the study. There was a significant decline in the bone resorption marker NTX-telopeptides in the alendronate group as compared to the placebo group at 18 months. There were no effects on the bone formation marker osteocalcin and bone pain. It is unknown whether decrease in bone turnover markers reflects the decline in metabolic activity of FD lesions [11]. The association between decrease in bone turnover markers and activity of the disease is not as clear as in the Paget disease. Decrement in bone turnover markers may be due to effect of bisphosphonates on the unaffected bone. Future studies including bone histomorphometry would allow more direct investigation of the effects of bisphosphonates on FD lesions versus unaffected bone. Bone turnover markers may not fit in normal reference range although improvement in clinical picture is provided. Overtreatment with bisphosphonates should be avoided.

In a case series of 26 patients with FD, 23 patients (89%) received treatment with bisphosphonates for a median 4 years (3–276 months) [6]. Different types of bisphosphonates were prescribed. At the end of follow-up, most patients were on treatment with zoledronic acid. Three patients reported pain relief with bisphosphonate treatment. Radiologic regression was observed in 2 of 18 patients who were followed up radiologically with bisphosphonate treatment. In the remaining 13 patients, there was no change in radiological scene. There was a progression in three patients [6].

As treatment option, surgery is indicated for the correction of deformities, management of fractures, and relieving the symptoms of compression [12]. One of our patients, P1, had surgery for recurrent femur fractures and craniofacial reconstructive surgery. Involvement of craniofacial site occurs in 25% of FD patients. Facial involvement can cause both facial asymmetry and compression for the anatomic structures located near to the affected bone [12]. Fractures are seen infrequently after puberty. In our P1, she had three times fractures till adulthood and there were no fractures after that.

## 5. Conclusions

Bisphosphonates may alleviate bone pain in fibrous dysplasia; however, the effect of medical therapy on skeletal destruction is not clear. In our cases of fibrous dysplasia, there was no change in radiological findings although there was reduction in bone turnover markers and symptoms. We recommend bisphosphonate treatment to fibrous dysplasia patients with symptom of bone pain according to our experience. These patients should be followed closely with radiological and laboratory data for long term complications and continuity of the treatment. Whether or not bisphosphonate slows the disease progression needs to be determined in controlled clinical trials.

## Figures and Tables

**Table 1:** Bone markers before and after bisphosphonate treatment.

Patient 1 (PFD)	ALP (U/L)	Ca (mg/dL)	P (mg/dL)	PTH (pg/mL)	OC (ng/mL)	DPD (nmol/L)	Dvit (nmol/L)
Before tx	975	9.6	3.4	81	50.7	83.1	56
After 6 months of 1st pamidronate tx	612	9.3	3.4	66	100	65.6	—
After 6 months of 2nd pamidronate tx	632	9.7	2.8	55	93.7	41.7	—
After 2 years	520	9.2	NA	64.98	92.9	38.55	—

Patient 2 (MFD)	ALP (U/L)	Ca (mg/dL)	P (mg/dL)	PTH (pg/mL)	OC (ng/mL)	DPD (nmol/L)	Dvit (nmol/L)

Before tx	54	10.5	4	20	10.8	5.45	24
After 12 months of 1st pamidronate tx	55	10	3.2	29	5.4	4.6	—

Patient 3 (MFD)	ALP (U/L)	Ca (mg/dL)	P (mg/dL)	PTH (pg/mL)	OC (ng/mL)	DPD (nmol)	Dvit (nmol/L)

Before tx	66	9.6	4.7	31	5.56	10.97	40
After 12 months of 1st zoledronic a. tx	84	10.1	4.3	37.6	3.97	8	—

Patient 4 (MFD)	ALP (U/L)	Ca (mg/dL)	P (mg/dL)	PTH (pg/mL)	OC (ng/mL)	DPD (nmol/L)	Dvit (nmol/L)

Before tx	111	9.6	3.2	32.7	25.6	11.43	31
After 12 months of 1st pamidronate tx	84	9.3	3.2	31.9	12.6	11.01	—

Patient 5 (PFD) (MAS)	ALP (U/L)	Ca (mg/dL)	P (mg/dL)	PTH (pg/mL)	OC (ng/mL)	DPD (nmol/L)	Dvit (nmol/L)

Before tx	227	10.5	3.9	54.5	15.8	8	29
After 3 months of 1st pamidronate tx	138	9.5	3.0	48	NA	5.2	—

**Table 2:** Characteristics of our patients with FD.

	Age, gender	Type	Clinical findings at the time of dx	Medical department first place of admission	Biopsy verified
P1	31 y, F	Polyostotic	Femur fracture	Emergency	Yes
P2	30 y, M	Monostotic	Bone pain	Plastic Surgery	No
P3	53 y, F	Monostotic	Headache	Neurosurgery	No
P4	43 y, F	Monostotic	Bone deformity	ENT^*∗*^	No
P5	43 y, M	Polyostotic	Bone deformity	Plastic Surgery	Yes

^*∗*^ENT: ear, nose, and throat.
